# Isolation and Characterization of a Biocontrol Serine Protease from *Pseudomonas aeruginosa* FZM498 Involved in Antagonistic Activity Against *Blastocystis* sp. Parasite

**DOI:** 10.3390/biom16010082

**Published:** 2026-01-04

**Authors:** Fatimah Z. Almilad, Essam Kotb, Hanadi B. Baghdadi, Nehal Hosin, Hawra A. Alsaif, Ayman A. El-Badry

**Affiliations:** 1 Department of Biology, College of Science, Imam Abdulrahman Bin Faisal University (IAU), P.O. Box 1982, Dammam 31441, Saudi Arabiahawra.ali.saif@gmail.com (H.A.A.); 2 Basic and Applied Scientific Research Center (BASRC), Imam Abdulrahman Bin Faisal University (IAU), P.O. Box 1982, Dammam 31441, Saudi Arabia; 3 Department of Microbiology, College of Medicine, Imam Abdulrahman Bin Faisal University (IAU), P.O. Box 1982, Dammam 31441, Saudi Arabia; nmhosin@iau.edu.sa (N.H.); or aelbadry@kasralainy.edu.eg (A.A.E.-B.)

**Keywords:** protease, *Blastocystis*, biological control, fecal bacteria

## Abstract

The intestine is considered a habitat for both bacteria and parasites. In this study, many fecal bacterial isolates and the protozoan *Blastocystis* sp. were recovered from stool samples of individuals with gastrointestinal conditions. Isolated bacteria were tested for extracellular protease production, and the most potent producer was identified by *16SrDNA* gene sequencing as *P. aeruginosa* FZM498. The enzyme was extracted and purified to electrophoretic homogeneity by the DEAE-Sepharose ion-exchanger and SDS-PAGE revealed a major band at 42.15 KDa. It exhibited maximal activity at 35 °C with thermostability at 60 °C (*T*_1/2_ = 200.04 min). It was most active at pH 8.0 and stable at 5.0–9.5. Enzymatic activity was greatly stimulated in the presence of Fe^2+^ ions, but was repressed by Zn^2+^ and Hg^2+^ ions. Inhibition by PMSF, TLCK, aprotinin, benzamidine, and SBTI protease reagents suggests a serine protease family. The *V*_max_ and *K*_m_ dynamic constants against azocasein were 36.232 U/mL and 0.0072 mM, respectively. It exhibited the lowest *K*_m_ value against the synthetic substrate D-Val-Leu-Lys-pNA among all substrates, indicating a plasmin-like activity. Interestingly, when tested against *Blastocystis* sp., cysts appeared progressively shrunken, ruptured, and mycelial-like, indicating complete structural collapse with leakage of intracellular contents. The importance of this research is that it is the first study to test the anti-*Blastocystis* activity of an extracted bacterial serine protease from the gut. This could be a promising, eco-friendly, natural alternative as an anti-*Blastocystis* agent. The objective of this study was to isolate, purify, and biochemically characterize an extracellular serine protease produced by gut-associated bacteria, as well as to assess its in vitro anti-*Blastocystis* efficacy as a potential natural and ecologically friendly antiparasitic therapy.

## 1. Introduction

The human intestine harbors a dynamic ecosystem of microorganisms, including bacteria, fungi, and eukaryotic parasites, the interactions of which are fundamental to maintaining intestinal homeostasis. Recent research has increasingly focused on the complex and often controversial relationship between *Blastocystis* sp., a common intestinal protist, and the gut microbiota. *Blastocystis* has been correlated to changes in the composition of gut bacteria, indicating that, depending on the parasite subtype, host immunological response, and the surrounding microbial environment, its biological behavior may vary from commensal to opportunistic [1].

Although numerous studies have explored the biology and pathogenic potential of *Blastocystis*, few have examined it in the context of human gut microbiota diversity [2]. Evidence suggests that *Blastocystis* colonization correlates with distinct bacterial signatures; individuals carrying the parasite tend to harbor higher levels of *Clostridiales vadin*BB60, whereas those without it exhibit greater abundances of *Bacteroidaceae* and *Escherichia–Shigella* [3]. Members of the Enterobacteriaceae family are of particular interest because of their known relationship with intestinal inflammation and dysbiosis. Certain *Blastocystis*-positive individuals have been found to have an increased prevalence of Enterobacteriaceae, which may create a pro-inflammatory gut environment that aids parasite persistence or exacerbates host reactions [2]. The frequent detection of *Blastocystis* in asymptomatic individuals has raised debate over its commensal versus pathogenic nature. Similar to *Escherichia coli*, the behavior of *Blastocystis*, whether benign or harmful, appears to depend on its pan-genomic diversity, host physiology, and the composition of the surrounding microbial ecosystem [4]. Consequently, the same organism may exist as a commensal in one host while acting as a pathogen in another, with host nutrition and immune status playing decisive roles [5].

From a clinical and public health standpoint, the interaction of *Blastocystis*, gut bacteria, and host immunity adds to the ongoing debate about the necessity for treatment. *Blastocystis* infection has a wide range of clinical presentations, from asymptomatic carriage to gastrointestinal symptoms. This heterogeneity is increasingly related to changes in gut microbiota composition and host immunological responses, highlighting the importance of evaluating *Blastocystis’* health impact in the context of host–microbiome–parasite interactions rather than in isolation [1].

Certain commensal bacteria, such as *Bacteroides* and *Prevotella*, can influence parasite colonization and virulence through the secretion of enzymes like proteases and glycosidases. These enzymes are capable of degrading mucins, modifying the intestinal environment, and thereby facilitating *Blastocystis* adherence and persistence on the mucosal surface [4,6]. Conversely, *Blastocystis* may also alter the composition of the gut microbiota by modulating immune responses or competing for nutrients, creating a bidirectional relationship. Moreover, microbial enzymes have been proposed to directly affect *Blastocystis* cyst wall integrity or interfere with its replication cycle, although these mechanisms remain under investigation [7,8].

Proteases represent one of the most functionally diverse enzyme classes, catalyzing peptide bond cleavage and contributing to a wide range of physiological and ecological processes. Within the gut, bacterial proteases are involved not only in digestion but also in regulating host–microbe and microbe–microbe interactions [9]. Proteases derived from the gut microbiota are of particular interest because they are naturally adapted to the same environmental conditions as intestinal protozoa, including *Blastocystis*. This ecological compatibility positions them as promising candidates for targeted biocontrol strategies.

Conventional antiprotozoal agents, such as metronidazole, are limited by variable efficacy and adverse effects, prompting the search for safer, microbiota-compatible alternatives. Bacterial proteases present an appealing option as their enzymatic activity could compromise parasite viability by degrading essential structural proteins or indirectly impeding colonization by altering mucosal adhesion or nutrient availability [10].

The present study investigates gut-derived bacterial protease as a potential biocontrol agent against *Blastocystis* sp. The objective is to evaluate its efficacy, enzymatic characteristics, and in vitro safety profile, providing a foundation for developing a natural, enzyme-based therapeutic alternative to traditional antiparasitic drugs. This approach aligns with the growing interest in sustainable, microbiome-oriented strategies for managing intestinal parasitic infections. The present study investigates gut-derived bacterial protease as a potential biocontrol agent against *Blastocystis* sp. The objective is to evaluate its efficacy, enzymatic characteristics, and in vitro safety profile, providing a foundation for developing a natural, enzyme-based therapeutic alternative to traditional antiparasitic drugs. This approach aligns with the growing interest in sustainable, microbiome-oriented strategies for managing intestinal parasitic infections.

## 2. Materials and Methods

### 2.1. Sample Processing and Isolation of Fecal Bacteria and Parasites

One hundred forty-four stool specimens were collected from individuals visiting King Fahd Hospital of Imam Abdulrahman Bin Faisal University in Al Khobar, Saudi Arabia. Each specimen was subdivided into three fractions. The first fraction (200 mg) was used for the isolation of *Blastocystis* parasites. It is cultured in Jones’ medium supplemented with 10% heat-inactivated horse serum and incubated in an anaerobic condition at 37 °C for 48 h. The positive samples were sub-cultured in IMDM medium with a penicillin (200 µg/mL) and streptomycin (200 µg/mL) solution as described by Karamati et al. [11]. It was microscopically examined daily with a 40× objective lens for the presence of *Blastocystis* sp.

The second fraction of the specimen (200 mg) was used for the isolation of enteric bacteria on McConkey agar. After 24 h of incubation at 35 °C, pure colonies were prepared by quadrate streaking on the same medium, then preserved at 4 °C for short-term usage. The third fraction of stool samples (50 mg) was maintained at −80 °C for DNA extraction. *Blastocystis*-DNA amplification was carried out in a thermocycler using the PCR cycles and conditions described by Clark [12] with minor changes. DNA amplified products were analyzed using 1.5% agarose gel electrophoresis and ultraviolet transillumination following staining with ethidium bromide.

### 2.2. Qualitative Protease Detection and Production

Preliminary test for extracellular protease productivity by bacterial isolates was detected by spotting skimmed-milk agar plates consisting of 15 g/L agar, 2 g/L meat extract, 1 g/L peptone, 5 g/L sodium chloride, 2 g/L yeast extract, 0.02 g/L methylene blue, and 250 mL of skim milk. The medium pH was adjusted to 7.0, and the positive hydrolytic colonies were visualized after 24 h of incubation at 37 °C. Pure cultures were preserved at −80 °C in 20% glycerol for future investigations.

Promising bacteria were cultivated in a basal medium to produce crude enzymes. This composed of 10 g/L soluble casein, 5 g/L glucose, 0.4 g/L yeast extract, 3 g/L sodium chloride, 2.2 g/L dipotassium phosphate (K_2_HPO_4_), 1 g/L monopotassium phosphate (KH_2_PO_4_), 0.5 g/L magnesium sulfate heptahydrate (MgSO_4_·7H_2_O), 0.1 g/L manganese sulfate heptahydrate (MnSO_4_·7H_2_O), 0.02 g/L ferrous sulfate (FeSO_4_), and 0.02 g/L of zinc sulfate (ZnSO_4_). The medium pH was adjusted to 7.0, and the fermentation process lasted for 48 h at 37 °C while being shaken at 200 rpm. Centrifugation was carried out using a centrifuge (HETTICH Centrifuge RIF 1406, Raptor Supplies, Manchester, UK) for 20 min at 5000 rpm to separate the bacterial cells at 4 °C. Supernatants were utilized to assess protease production [13].

### 2.3. Quantitative Assay of Protease Activity

Protease activity was measured by mixing 1 mL of 0.25% (*w*/*v*) azocasein solution in 0.2 M Tris–HCl buffer (pH 8.0) with 1 mL of crude protease. After allowing the enzyme–substrate reaction to proceed for 1 h at 37 °C, 2 mL of 10% (*w*/*v*) TCA solution was added to stop the reaction. The mixture was then incubated in a bath of crushed ice for 60 min. The amount of soluble degradation proteins (C) (mg/mL) was determined using the following calculation: C (mg/mL) = 1.55 *A*_280_ − 0.76 *A*_260_ [14].

### 2.4. Identification of Bacteria and Parasites

The purified *16SrDNA* gene products from the selected bacterium were sequenced with the same forward (5′-AGAGTTTGATCCTGGCTCAG-3′) and reverse (5′-TACGGCTACCTTGTTACGACTT-3′) primers separately using Applied Biosystems 3500 genetic analyzers (Applied Biosystems, Foster City, CA, USA). The cycle sequencing reaction was carried out using BigDye^®^ Terminator v3.1 Cycle Sequencing Kit for cycle sequencing and purification of cycle sequencing product (Applied Biosystems, Foster City, CA, USA).

*Blastocystis* cysts were identified microscopically after staining with trichrome to identify the internal structure. It was identified molecularly using PCR following Stensvold [15]. One mL of positive subculture for *Blastocystis* sp. was suspended in PBS, then centrifuged at 12,000× *g* for one min to pellet the *Blastocystis* sp. cysts. Genomic DNA was extracted from pelleted *Blastocystis* sp. cysts using the commercial DNA extraction kit from Zymo Research (Irvine, CA, USA) according to the kit’s procedure. *Blastocystis* DNA was amplified using primers (The RD5: 5′-ATCTGGTTGATCCTG CCAGT-3′ and BhRDr: 5′-GAGCTTTTTAACTGC AACAACG-3′) targeting *Blastocystis* species-specific SSU rDNA. PCR reaction conditions were utilized according to Scicluna et al. [16].

### 2.5. Purification of Bacterial Protease

This was performed through multiple stages comprising ethanol precipitation, dialysis, and ion exchange chromatography. Fermentation broth was separated from the bacterial cells by centrifugation at 5000 rpm for 20 min, then soluble proteins were salted out by gradual addition of cold ethanol with vigorous stirring till 60% (*v*/*v*) concentration limit. The enzyme was dialyzed to remove the nonprotein structures. The dialysis tube was put in 250 mL of 500 mM chilled sodium borate buffer (pH 8.6) with stirring at 4 °C for 12 h. The dialyzed free proteins were eluted at 1 mL/min through a DEAE-Sepharose column (1 × 15 cm) pre-equilibrated using 50 mM Tris-HCl buffer (pH 7.0, buffer A). The NGC system (Bio-Rad, Hercules, CA, USA) was used in the separation process. However, the bounded proteins were eluted with 50 mM Tris-HCl buffer (pH 8.5) containing 0.42% (*v*/*v*) acetone with a linear rise in NaCl strength from 20 mM to 800 mM (buffer B). Fractions were collected based on absorbance readings at 280 nm and evaluated for enzymatic activity. Active fractions were pooled, lyophilized, and kept at 4 °C for characterization studies. SDS-PAGE was performed to estimate the enzyme’s molecular mass and homogeneity, utilizing 4% stacking gel and 12% resolving gel at a constant voltage of 200 V. Protein bands were stained by immersion in Page Blue for 5 min and destained by soaking in dH2O for another 5 min [17].

### 2.6. Effect of Temperature on Protease Activity and Stability

To determine the enzyme’s optimal temperature, the enzyme–substrate combinations were incubated for 60 min across 20–55 °C at pH 7.0, and the amount of protein product was quantified. Thermal stability was designed to determine the range of temperature within which the secreted protease maintained its activity. The enzyme was incubated in a water bath for 15, 30, 45, and 60 min at varying temperatures, namely 50, 60, 70, and 80 °C. At the end of incubation, the enzyme was poured into an assay medium at 35 °C for 1 h. Protease activity only was determined, as previously mentioned. Relative activity is expressed as the percentage of the maximum activity (100%) under standard assay conditions. The unincubated enzyme served as a positive control. The half-life time (*T*_1/2_) was then calculated [18].

### 2.7. Effect of pH on Enzymatic Activity and Stability

Enzyme activity was tested at different pHs from 5 to 12 in (20 mM sodium acetate buffer (pH 5.0–6.0), 20 mM sodium phosphate buffer (pH 6.5–8.0), and 20 mM glycine–NaOH buffer (pH 8.5–12.0) by incubating crude enzyme–substrate under standard assay conditions. Experiments were carried out utilizing the standard protease assay with azocasein (0.25%, *w*/*v*) as substrate and incubation at 35 °C for 1 h. As previously stated, relative activity was determined. For the pH stability experiment, preincubation of buffered enzyme fractions without substrate at 35 °C for 1 h in pH 5–12 was performed, then relative activity was determined at pH 8.0 and 35 °C.

### 2.8. Effect of Cations

Protease stability was examined in parallel experiments in the presence of Co^2+^, Zn^2+^, Hg^2+^, Ca^2+^, Fe^2+^, Na^+^, Ba^2+^, Mn^2+^, Ni^2+^, Mg^2+^, K^+^, and Mg^2+^ ions. The enzyme was incubated at 35 °C for 1h with each metal ion at a final concentration of 5 mM. The mixture was then incubated with 1 mL of 0.25% (*w*/*v*) of azocasein as a substrate in 0.5 M Tris buffer (pH 8.0) at 35 °C for 1 h, and relative activity was measured after stopping the reaction with TCA solution. The activity rate was determined as a percentage of the relative activity (with metal ions) of the control activity (without metal ions).

### 2.9. Inhibition Study

The impact of common protease inhibitors on the enzyme activity was studied using PMSF (5 mM, 2 mM.), EDTA (10 mM), DMSO (10 mM), 1,10-phenoathroline (0.1 mM), 2,2-bipyridyl (0.1 mM), 1,4-DTT (50 mM, 10 mM), pepstatin (0.05 mM, 0.02 mM), leupeptin (0.05 mM, 0.02 mM), and 2-mercaptoethanol (50 mM, 10 mM). The reaction was performed at 35 °C for 1 h, then residual activity was started by adding 0.25% azocasein as a substrate. The rate of inhibition was established as a percentage of the residual activity (with inhibitor) of the control activity (without inhibitor) [19].

### 2.10. Determination of Kinetic Parameters

This was performed to assess the dynamics of the extracted protease. The effect of increasing substrate (azocasein) concentration (0.005–0.010%) on the reaction rate was measured at pH 8.0 and 35 °C, then enzymatic activity was determined as above. The *K*_m_, *V*_max_, and *K*_cat_ were evaluated. *K*_m_ and *V*_max_ were calculated by linear regression from the LB plot.

### 2.11. Amidolytic Activity 

This was determined spectrophotometrically using synthetic chromogenic substrates, including S-6258, S-2288, S-7388, and S-7127. The assay was performed by mixing 5 mg of the purified enzyme with 200 µL of a 0.2 mM chromogenic substrate solution. The reaction was continuously monitored for 5 min at 37 °C using a temperature-controlled spectrophotometer. The amount of released pNA was quantified by recording the change in absorbance at 405 nm.

### 2.12. Anti-Blastocystis Activity of Bacterial Protease

The target protease was tested as a biological control agent against *Blastocystis*. A suspension of cultured *Blastocystis* sp. in Jones’ culture medium, in the logarithmic growth phase of 10^6^/mL, was inoculated in a microplate assay (150 µL). Each well of the microplate contained 150 µL of sterile distilled water to perform two-fold serial dilutions. The assay included three groups, each in triplicate: (1) negative control, wells containing cultured *Blastocystis* cysts, (2) positive control, wells containing cultured *Blastocystis* cysts exposed to 500 μg/mL metronidazole, which is a reference anti-*Blastocystis*, and (3) experiment, each well containing cultured *Blastocystis* cysts exposed to protease (50 µg/mL). After inoculation, the microplate was incubated under anaerobic conditions at 37 °C for 12 h and was examined at hourly intervals for *Blastocystis* cysts viability [20].

#### 2.12.1. Light Microscopy

The viability of *Blastocystis* was assessed by light microscope using a 0.4% trypan blue solution. A sample from each well was mixed with the dye and examined microscopically. Viable cysts excluded the dye and appeared clear, while non-viable cysts absorbed the dye and stained blue. The number of live and dead cysts was counted to determine the viability percentage at each time point [20].

#### 2.12.2. Scanning Electron Microscopy

The harvested cysts after enzymatic treatment were resuspended in 2.5% (*v*/*v*) glutaraldehyde solution prepared in cacodylate buffer to preserve the cellular structures. This fixation is typically carried out for 2 h at 4 °C. The cysts were then washed with 0.2 M sodium phosphate solution buffer (pH 7.4) to remove any excess fixative, minimizing the risk of artifacts during imaging. Next, secondary fixation was performed using 2% osmium tetroxide, which further stabilizes the ultrastructure and typically lasts for 2 h at room temperature. Once fixation is complete, the sample undergoes dehydration through a graded series of ethanol or acetone concentrations (30%, 50%, 70%, 90%, and 100%). After dehydration, drying is carried out using the critical point drying method. Finally, the dried sample is coated with a thin layer of gold, using a sputter coater to enhance conductivity and improve imaging quality under the electron microscope (JSM- 6390LA, JEOL Ltd., Tokyo, Japan) [21].

### 2.13. Statistical Analysis

The raw results were statistically analyzed with SPSS software (Version 31) and reported as means ± standard deviations. Each reading is the mean of three independent tests. Means were compared, variances were examined, and *p*-values less than 0.05 were an indication of statistical significance.

## 3. Results

### 3.1. Isolation and Identification of Fecal Bacteria and Parasites

**Figure 1:**
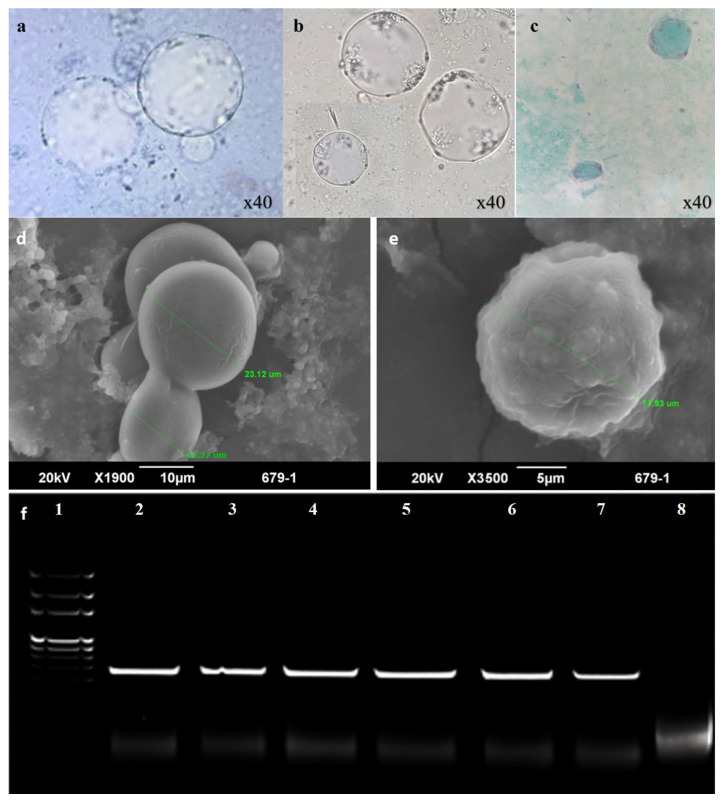
Light Microscope images of isolated *Blastocystis* ((**a**) unstained, (**b**) iodine-stained, (**c**) trichrome-stained). *Blastocystis* was also visualized under SEM (**d**). Cultured *Blastocystis* sp. undergoing binary fission under SEM (**e**). Panel (**f**). represents agarose gel electrophoresis for PCR amplifying *Blastocystis* species-specific SSU rDNA. Lane (1): 100 bp DNA marker. Lane (2): Positive control sample of *Blastocystis* species. Lane 8: Negative control. Lanes (3–7) represent positive results for *Blastocystis* species.

Out of 144 fecal samples collected from people with gastrointestinal disorders using Jones’ medium, *Blastocystis* sp. were identified in 18 (12.5%) samples. The isolates were subcultured in IMDM using a solution of penicillin and streptomycin. When examined under LM, *Blastocystis* sp. appeared with a clearly defined central vacuole, surrounded by granular cytoplasm typical of the vacuolar stage commonly seen in stool specimens (Figure 1a–c). SEM examination also confirmed the identity of *Blastocystis* sp. (Figure 1d,e). (Figure 1f) Genetic identification was performed using PCR amplification of the conserved *Blastocystis* species-specific 100 bp target of the SSU rRNA gene. 

**Figure 2:**
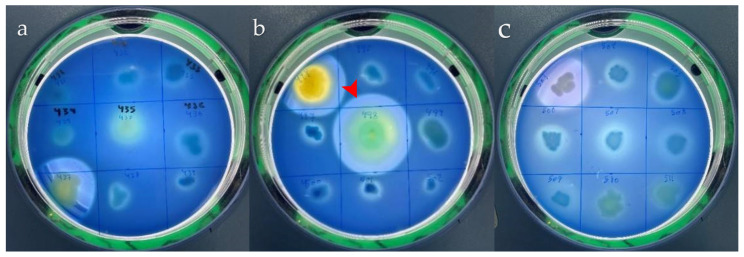
Screening of protease secreted by fecal bacteria on skimmed milk agar, after 48 h of incubation at 35 °C (panels (**a**–**c**)). We have obtained so many bacteria, among these, isolate 498 was found to be the most potent producer, with a large clear zone and maximum activity (panel (**b**), red arrowhead).

**Figure 3:**
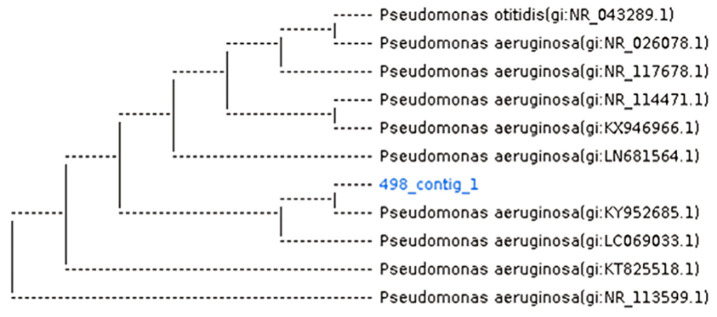
Phylogenetic tree of the selected isolate. The *16SrDNA* gene sequence was deposited in the GenBank database under accession number PX497606. The blasted phylogenetic tree shows identity with *P. aeruginosa*, and the strain was designated as FZM498.

In parallel, enteric bacteria were isolated from the same stool samples and screened for their secreted protease activity to select a strain with the highest enzymatic activity. Results in Figure 2 show clear zones on skimmed milk agar with different halos due to the degradation of skimmed milk by the proteolytic activity. Isolate 498 was found to display the highest proteolytic activity compared to others (Figure 2b, red arrowhead). The most potent isolate was identified, and the *16SrDNA* gene sequence was deposited in the GenBank database under accession number PX497606. The blasted phylogenetic tree (Figure 3) shows identity with *P. aeruginosa*, and the strain was designated as FZM498.

### 3.2. Purification of Selected Protease

**Figure 4:**
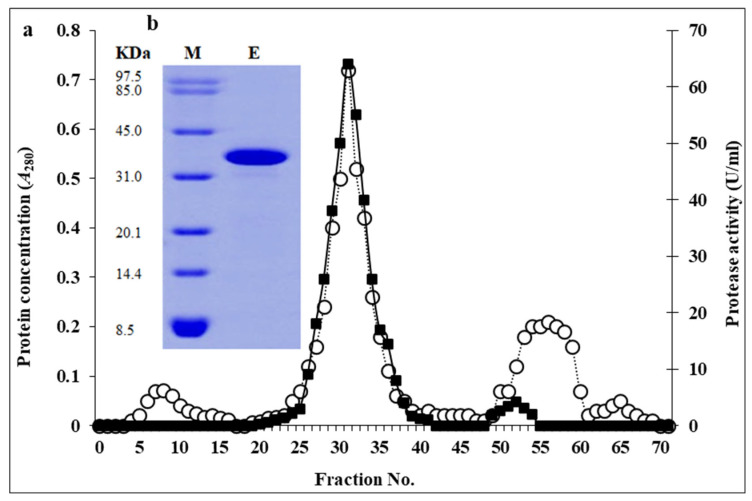
Panel (**a**) represents the elution profile of the tested protease through a DEAE-Sepharose column (1 × 15 cm). Enzyme activity (-■-) and protein content (-○-). Panel (**b**) represents SDS-PAGE of the purified enzyme, utilizing 4% stacking gel and 12% resolving gel at a constant voltage of 200 V. Protein bands were stained by immersion in PageBlue for 5 min and destained by soaking in dH_2_O for another 5 min. Lane M represents protein markers, and lane E represents the purified enzyme.

The enzyme was mass-produced in the basal medium described in Section 2. It was then precipitated by 60% (*v*/*v*) ethanol and dialyzed to remove the nonprotein structures. The dialysate eluted through a DEAE-Sepharose column (1 × 15 cm) shows an active protease peak between fraction 24 and fraction 38, and the most active fraction was number 31 (Figure 4a). This active peak was tested for homogeneity and molecular weight determination by SDS-PAGE analysis. A major band at 42.145 KDa (Figure 4b) was observed, which is indicative of the molecular mass of the protease under study.

### 3.3. Effect of Temperature and pH on Protease Activity and Stability

**Figure 5:**
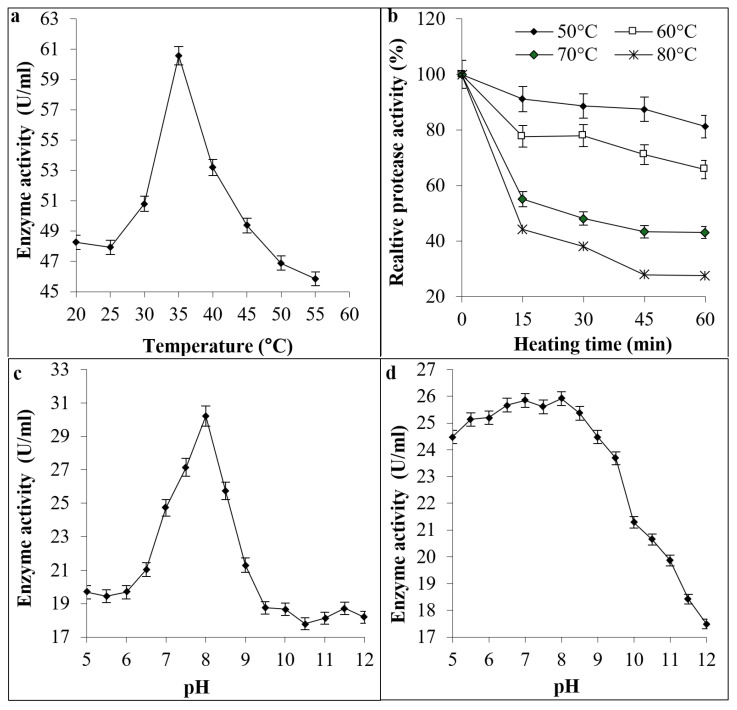
Effect of temperature on activity (**a**) and stability (**b**) of bacterial protease. In addition, the effect of pH on the activity (**c**) and stability (**d**) is shown.

The tested protease showed maximum activity at 35 °C (Figure 5a). Furthermore, it retained thermal stability till 60 °C for 60 min. At 70 °C, activity declines more dramatically, dropping roughly to 40% after 60 min, demonstrating partial thermal denaturation over time (Figure 5b). The results shown in Figure 5c declare that pH 8.0 is optimal for maximum protease activity. In addition, protease stability was maximum at pH 5.0–9.5 (Figure 5d).

### 3.4. Effect of Metallic Ions

**Figure 6:**
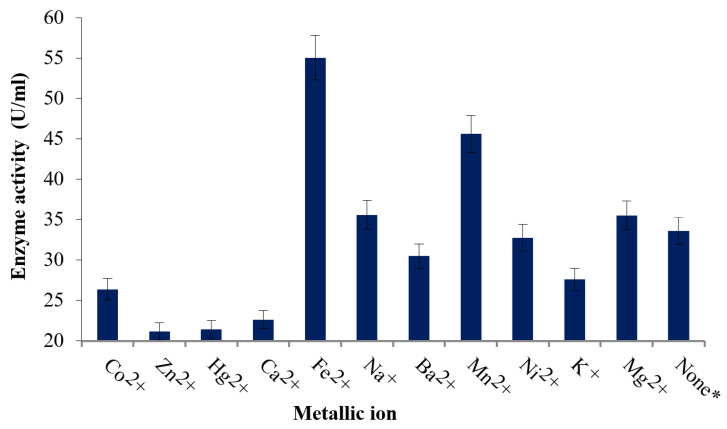
Effect of metallic ions on protease activity. The relative activity was calculated in comparison with treatment without any metal ions (*, 100% activity).

Figure 6 represents the effect of Co^2+^, Zn^2+^, Hg^2+^, Ca^2+^, Fe^2+^, Na^+^, Ba^2+^, Mn^2+^, Ni^2+^, Mg^2+^, K^+^, and Mg^2+^ ions on the activity of selected protease. In all treatments, a control sample was run simultaneously, parallel to each treatment of the enzyme under study. The highest relative activity was found in the presence of Fe^2+^ ions (163.81%), followed by Mn^2+^ ions (163.81%). However, enzymatic inhibition was found in the presence of Hg^2+^ ions (63.80%) and Zn^2+^ ions (63.02%).

### 3.5. Effect of Protease Inhibitors

The protease activity was inhibited by many reagents; high sensitivity was demonstrated by PMSF, which dramatically reduced enzyme activity to <10% at both 2 and 5 mM concentrations. Aprotinin, SBTI, benzamidine, and TLCK also drastically decreased enzyme activity. However, EDTA, 1,10-phenanthroline, and 2,2-bipyridine did not inhibit the enzyme activity. Furthermore, the enzyme does not belong to the groups of proteases that were inhibited by leupeptin and E-64 (cysteine protease inhibitors) and pepstatin A (aspartic protease inhibitor), which demonstrated weak inhibition. Reducers such as 1,4-DTT and 2-mercaptoethanol lowered activity (Table 1).

### 3.6. Enzyme Dynamics

**Figure 7:**
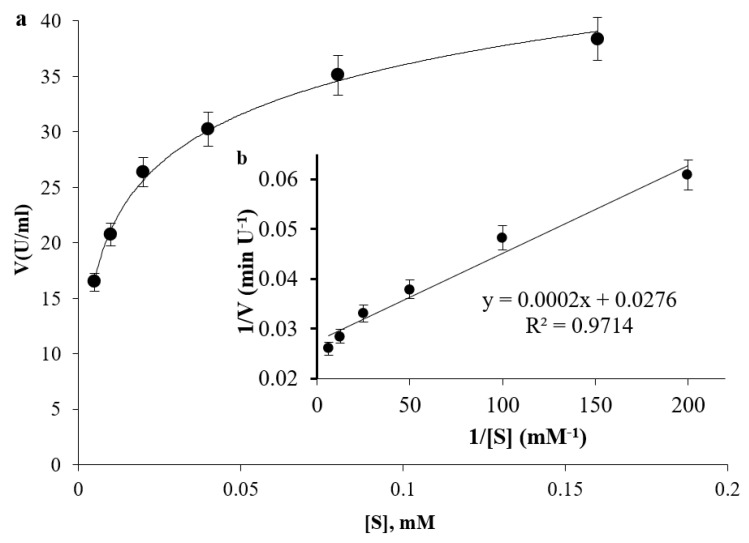
Effect of azocasein concentrations on enzyme activity in the form of a hyperbolic plot (**a**) and LB plot (**b**).

The tested protease showed a hyperbolic pattern against increasing concentrations of the substrate azocasein (Figure 7a). The reciprocal of parameters was plotted in the form of an LB plot (Figure 7b) to deduce the maximum rate of reaction (*V*_max_) and the Maechalis constant (*K*_m_). The apparent *V*_max_ was 36.232 U/mL, and the *K*_m_ was 0.0072 mM.

### 3.7. Substrate Specificity

Considering each substrate is associated with a recognized protease class, such as kallikrein, tPA, subtilisin, and plasmin, it is possible to determine the enzyme’s preferred substrate and possible classification. All substrates exhibit classic Michaelis–Menten behavior, according to the kinetic data, with increased activity as substrate concentration rises. However, S-7127 (D-Val-Leu-Lys-pNA) gave the lowest *K*_m_ value (0.005128 mM), suggesting the strongest substrate affinity, and the class to which it belongs (Table 2).

### 3.8. In Vitro Anti-Blastocystis Activity of Bacterial Protease

**Figure 8:**
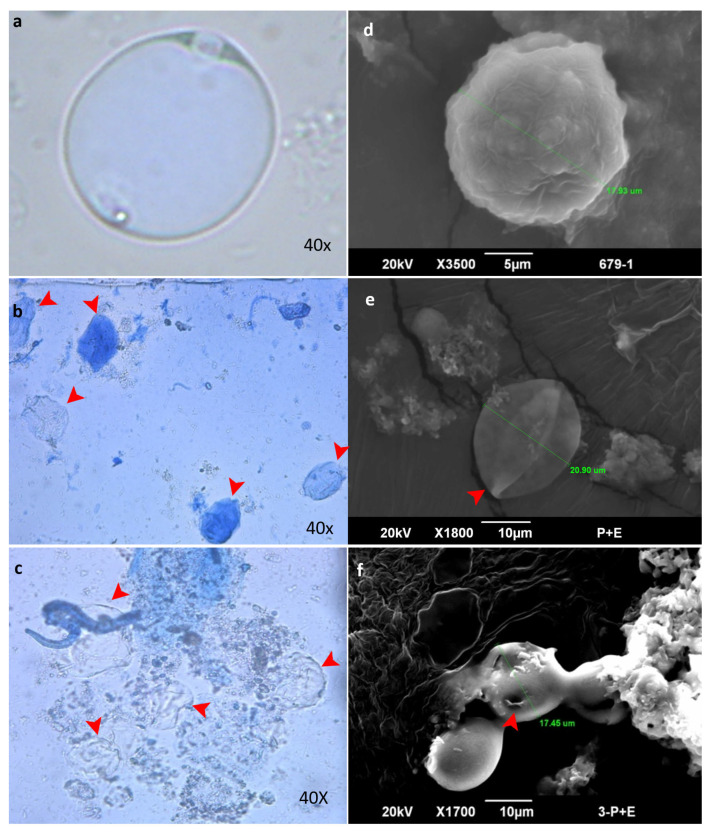
Cells of *Blastocystis* sp. treated by bacterial protease at 50 µg/mL concentration. Panels (**a**,**d**) represent the untreated cells under LM and SEM, respectively. Cysts stained by trypan blue under the Light microscope (**a**–**c**) show dead *Blastocystis* cysts ((**b**,**c**) red arrow heads). Captured SEM micrographs (**d**–**f**) confirm cell shrinkage, collapse after 2 h of exposure (**e**), and extensive damage with perforations after 4 h (**f**).

Immediately after 2 h of exposure to the bacterial enzyme, parasite cells displayed visible symptoms of stress (Figure 8). They appeared shrunken, with a noticeable size reduction, which suggested that the cellular damage had progressed (Figure 8b,e). The cells began to lyse after the fourth hour of exposure, and many of them became ruptured, expelling their contents (Figure 8c,f). These findings support that the purified bacterial enzyme from this study has the potential to be a biological control agent by time-dependently compromising the integrity of *Blastocystis* cells. The captured SEM micrographs revealed the extensive cellular damage, including deformation (Figure 8e) and perforations (Figure 8f), confirming the significant structural compromise observed under LM (Figure 8e,f).

## 4. Discussion

The human gut hosts a highly diverse microbiota composed of bacteria, protozoa, and other microorganisms that play pivotal roles in maintaining intestinal homeostasis. Among these, *Blastocystis* sp. exhibits a complex duality, functioning as either commensals or opportunistic pathogens depending on their genomic variability, the host’s physiological state, and the surrounding gut ecosystem [4,5]. The present study aimed to isolate and characterize bacterial strains producing extracellular proteases from fecal samples and to evaluate their potential biocontrol activity against *Blastocystis* sp.

According to the current study, the overall rate of *Blastocystis* infection was 12.5%. The current finding is consistent with previously reported prevalence rates in Iran (14.35%) [22] and Spain (9.18%) [23]. On the contrary, Brazil had slightly higher infection rates (24%) [24].

The PCR technique used in this study successfully amplified the predicted fragment, confirming the presence of *Blastocystis* DNA in all microscopically positive samples and establishing the molecular approach’s accuracy. This outcome aligns with earlier research, which demonstrates that PCR enhances detection sensitivity and frequently identifies illnesses that traditional microscopy may miss. For example, greater PCR detection rates were reported in Egypt [25] and in the UK [26].

Initial screening for protease production was conducted using skimmed milk agar, a casein-rich medium commonly used to detect extracellular proteolytic activity. Out of 144 isolates, several demonstrated protease secretions, with the most potent enzyme producer identified as *P. aeruginosa* FZM498. Protease detection using skimmed milk agar supplemented with methylene blue was particularly effective, providing a clear visual contrast between hydrolyzed and non-hydrolyzed zones. This colorimetric method proved to be rapid, sensitive, and non-inhibitory to bacterial growth, making it valuable for high-throughput screening of microbial proteases [27].

The purified protease from *P. aeruginosa* FZM498 displayed a molecular mass of approximately 42 kDa, which is consistent with the protease Prot 1 from *Botrytis cinerea* 43 kDa [28], and within the general range of bacterial serine proteases. Comparable molecular weights have been reported for proteases from *Brevibacillus agri* SAR25 50 kDa [29], and *B. subtilis* D9 48 kDa [30]. These similarities indicate that the enzyme belongs to the medium-molecular-weight class of bacterial proteases, often characterized by broad substrate specificity and robust catalytic efficiency.

Enzymatic characterization revealed that the FZM498 protease exhibited optimal activity at pH 8.0 and retained stability between pH 5.0 and 9.5. Comparable results were reported for *Halobacillus* sp. MBRI7 protease pH 7.0 [31], while other bacterial species showed broader alkaline stability, such as *B. halotolerans* DS5 pH 7.0–12.0 [32]; and *Alcaligenes faecalis* P2 pH 3.0–12.0 [33]. These findings highlight the adaptive flexibility of bacterial proteases toward diverse pH environments, reinforcing their suitability for potential biomedical applications.

Temperature also markedly influenced the catalytic performance of the enzyme. The optimal temperature for proteolytic activity was 35 °C, similar to that reported for *P. aeruginosa* MCMB-327 [34]. The enzyme remained stable up to 60 °C for 60 min, with a half-life (*T*_1/2_) of approximately 200 min, indicating significant thermostability. Comparable findings were observed in proteases from *B. amyloliquefaciens* HM48 [35] and *B. stearothermophilus* [36], suggesting that moderate thermostability is a common characteristic of mesophilic bacterial proteases. Such stability is advantageous for potential therapeutic or industrial applications, where enzyme resilience is critical.

Metal ion profiling demonstrated that Fe^2+^ and Mn^2+^ significantly enhanced protease activity, whereas Zn^2+^ and Hg^2+^ acted as strong inhibitors. This pattern aligns with earlier findings in *B. subtilis* PE-11, where divalent cations such as Ca^2+^, Mg^2+^, and Mn^2+^ activated the enzyme, while heavy metals like Hg^2+^ and Zn^2+^ suppressed activity [37]. The stimulatory effect of Fe^2+^ and Mn^2+^ observed in the present study suggests that these ions may play structural or catalytic roles in stabilizing the enzyme–substrate complex.

Inhibitor assays revealed that enzyme activity was completely abolished by PMSF, TLCK, aprotinin, benzamidine, and SBTI, confirming that the enzyme belongs to the serine protease family. The absence of inhibition by EDTA, 1,10-phenanthroline, and 2,2-bipyridine excluded the possibility of it being a metalloprotease. Similarly, weak inhibition by leupeptin, pepstatin A, and E-64 indicated that the enzyme is not of the cysteine or aspartic type. Reducing agents such as 1,4-DTT and 2-mercaptoethanol partially decreased activity, likely due to the disruption of disulfide bonds. Collectively, these findings clearly categorize the FZM498 enzyme as a serine protease, in agreement with similar observations reported by [38,39].

Kinetic studies revealed a hyperbolic Michaelis–Menten relationship, with a *V*_max_ of 36.23 U/mL and a *K*_m_ of 0.0072 mM for azocasein, suggesting high substrate affinity. Substrate specificity assays indicated significant activity against synthetic peptides N-Succinyl-Ala-Ala-Pro-Phe-pNA (S-7388) and D-Val-Leu-Lys-pNA·2HCl (S-7127), with the lowest *K*_m_ observed for S-7127 (0.0051 mM). This suggests a plasmin-like proteolytic mechanism, similar to the chymotrypsin-like serine proteases previously reported from *Bacillus*, *Vibrio*, and *Streptomyces* species [40,41,42].

The isolation of *Blastocystis* sp. presented several technical challenges due to the organism’s polymorphic forms and variable growth requirements. In this study, *Blastocystis* was successfully isolated under anaerobic conditions using Jones’ medium and subsequently subcultured in IMDM supplemented with antibiotics. Previous studies have employed comparable methods with variations in medium composition and atmospheric conditions [11,43], emphasizing the importance of optimizing culture parameters for consistent parasite recovery.

Biological control strategies are increasingly recognized as eco-friendly alternatives to conventional chemotherapeutics. The use of microbial enzymes, particularly proteases, offers a targeted approach to disrupting parasite structure and viability while minimizing ecological impact and the development of resistance. In the current study, the protease from *P. aeruginosa* FZM498 induced pronounced morphological damage to *Blastocystis* cysts in vitro. Within two hours of exposure, cells appeared shrunken and deformed, progressing to rupture and disintegration after four hours, indicative of enzymatic degradation of the cyst wall and subsequent cytoplasmic leakage.

Comparable antiparasitic effects have been reported using natural and nanoformulated agents. For instance, ethanolic extracts of *Artemisia judaica* inhibited *Blastocystis* growth by over 99% at 2000 µg/mL [44], while extracts of *Ceratonia siliqua* achieved more than 80% inhibition at 5 mg/mL [45]. Similarly, chitosan nanoparticles and antioxidant-loaded ZIF-8 composites exhibited concentration-dependent anti-*Blastocystis* activity, often surpassing metronidazole in efficacy [20,46]. In contrast, the FZM498 protease achieved parasite disruption at a markedly lower concentration (50 µg/mL), demonstrating high potency and biocompatibility potential.

## 5. Conclusions

In summary, this study reports the isolation, purification, and characterization of a serine protease from *P. aeruginosa* FZM498 exhibiting potent in vitro anti-*Blastocystis* activity. To the best of our knowledge, this is the first report describing a gut-derived bacterial protease with such biocontrol potential against *Blastocystis*. The findings highlight the enzyme’s stability, substrate affinity, and biological efficacy, suggesting its promise as a natural and environmentally safe antiparasitic agent.

## Figures and Tables

**Table 1:** Effect of various protease inhibitors on enzyme activity, measured by absorbance at 405 nm and expressed as relative activity (%) compared to the control (no inhibitor, 100%).

Protease Reagent	Standard Concentration (mM)	Enzyme Activity (*A*_405_)	Relative Activity (%)
PMSF	5.00	0.033	9.218
PMSF	2.00	0.034	9.497
TLCK	0.50	0.125	34.916
Aprotinin	0.05	0.236	65.922
SBTI	0.05	0.168	46.927
Benzamidine	1.00	0.254	70.950
EDTA	10.00	0.477	133.240
1,10-phenanthroline	0.10	0.536	149.721
2,2-bipyridine	0.10	0.509	142.179
1,4-DTT	50.00	0.198	55.307
1,4-DTT	10.00	0.254	70.950
Pepstatin A	0.05	0.473	132.123
Pepstatin A	0.02	0.382	106.704
Leupeptin	0.05	0.500	139.665
Leupeptin	0.02	0.354	98.883
2-mercaptoethanol	50.00	0.240	67.039
2-mercaptoethanol	10.00	0.341	95.251
E-64	1.50	0.353	98.603
None *	NA	0.358	100.000

*, absence of inhibitor. NA, not applicable.

**Table 2:** Substrate specificity with kinetic analysis.

Concentration (mM)	D-Val-Leu-Arg-P-Nitroanilide (S-6258)	D-Ile-Pro-Arg-AMC (S-2288)	N-Succinyl-Ala-Ala-Pro-Phe-P-Nitroanilide (S-7388)	D-Val-Leu-Lys-P-Nitroanilide Dihydrochloride (S-7127)
**0.200**	0.689	0.730	0.787	0.796
**0.100**	0.745	0.715	0.733	0.754
**0.050**	0.671	0.662	0.694	0.729
**0.025**	0.575	0.567	0.567	0.674
**0.000**	0.362	0.362	0.362	0.362
**Classification**	For Kallikrein	For tissue plasminogen-activator (tPA)	For subtilisin and chymotrypsin	For plasmin
***V*** **_max_**	66.23	61.73	66.67	64.10
***K*** **_m_**	0.013245	0.00617	0.01333	0.005128

## Data Availability

The datasets in this research can be found in online repositories. The names of the repositories and accession numbers are included in this article/Supplementary Material.

## Supplementary Materials

The following supporting information can be downloaded at: https://www.mdpi.com/article/10.3390/biom16010082/s1.

## Abbreviations

The following abbreviations are used in this manuscript:

DMSO	Dimethyl sulfoxide
1,4-DTT	1,4-dithiothreitol
EDTA	Ethylenediamine-tetraacetate
HMDS	Hexamethyldisilazane
IMDM	Iscove’s modified Dulbecco’s medium
*K*_cat_	Catalytic rate constant
K_m_	Michaelis–Menten constant
LB plot	Lineweaver–Burk plot
PBS	Phosphate-buffered saline
PMSF	Phenylmethyl sulfonyl fluoride
pNA	p-nitroaniline
SBTI	Soybean trypsin inhibitor
SDS-PAGE	Sodium dodecyl sulfate–polyacrylamide gel electrophoresis
*T*_1_/_2_	Half-life time
TCA	Trichloroacetic acid
TLCK	Tosyl-L-lysyl-chloromethane hydrochloride
V_max_	Maximum velocity of reaction
