# Supplementary figures and images for: Isolation and Characterization of a Biocontrol Serine Protease from Pseudomonas aeruginosa FZM498 Involved in Antagonistic Activity Against Blastocystis sp. Parasite
Source: Biomolecules. 2026 Jan 4;16(1):82. doi: 10.3390/biom16010082 (PMC12838579; doi:10.3390/biom16010082)

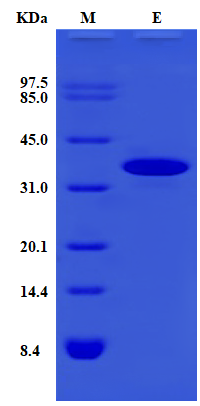

Supplement: Supplementary file 1 [file biomolecules-16-00082-s001.zip › biomolecules-4024363-supplementary.png]
